# Simplified computational model for generating biological networks†

**DOI:** 10.1039/d0ra06205g

**Published:** 2020-10-16

**Authors:** Matthew H. J. Bailey, David Ormrod Morley, Mark Wilson

**Affiliations:** Department of Chemistry, Physical and Theoretical Chemistry Laboratory, University of Oxford South Parks Road Oxford OX1 3QZ UK mark.wilson@chem.ox.ac.uk

## Abstract

A method to generate and simulate biological networks is discussed. An expanded Wooten–Winer–Weaire bond switching methods is proposed which allows for a distribution of node degrees in the network while conserving the mean average node degree. The networks are characterised in terms of their polygon structure and assortativities (a measure of local ordering). A wide range of experimental images are analysed and the underlying networks quantified in an analogous manner. Limitations in obtaining the network structure are discussed. A “network landscape” of the experimentally observed and simulated networks is constructed from the underlying metrics. The enhanced bond switching algorithm is able to generate networks spanning the full range of experimental observations.

## Abstract 2

We discuss a Monte Carlo method to simulate biological networks and compare to the underlying networks in experimental images.

## Graphical abstract

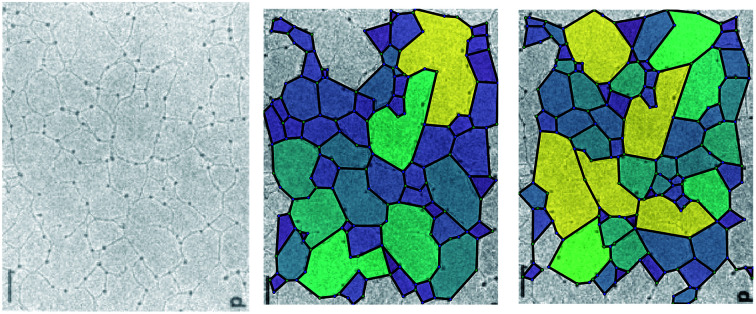

Two dimensional random networks are observed in a range of contexts across considerably different length scales in nature: from nanometres, in the form of amorphous graphene; to metres, in the form of the Giant's causeway; to tens of kilometres, in the form of geopolitical borders.^1–3^ A framework for describing these continuous random networks for chemical systems was first introduced by Zachariasen to describe silica-like glasses, and has proved to be extremely versatile in the years since,^4^ being used to describe biological networks, including actin networks and basement membranes made of collagen and laminin.^5–8^

Despite the physical diversity of continuous random networks, they share some common properties which arise from the fact that all planar 2D networks can be represented as a tiled arrangement of polygons, joined at their edges.^9^ Viewing a network as a collection of polygons explains some restrictions on the nature of the network. For example, the angles at each node must add up to 360° and the mean number of edges connected to each node affects the mean number of edges [or ring size] of each polygon. Traditionally, semi-empirical laws provided a theoretical understanding of the complex behaviour of real networks, most commonly for inorganic networks such as MgO grains, silica, or epithelial cells.^10–12^ Some examples of these laws include: Lewis' law,^12^ governing the distribution of polygon areas; Lemaître's law,^13^ relating the number of hexagons to the width of the polygon distribution; and the Aboav–Weaire law,^14^ governing the preference of polygons to be surrounded by similar or dissimilar polygons. Recent work has helped clarify the latter by re-casting the problem in terms of the assortativity, a metric designed for network theorists to describe social networks.^15,16^

Studying generic 2D networks in biology is a potentially difficult task. There are a variety of different techniques used, including fluorescence light-, atomic force and electron-microscopy.^17–19^ The resolution of light microscopy is limited by diffusion,^18^ and the power of electron or laser beams have to be limited to avoid damaging the delicate networks.^17^ However, even when a high-quality image is obtained, there is further difficulty in analysis. For example, each edge in a network is a complex molecule made up of tens of thousands of atoms which may not lie strictly in a single plane. In addition, the most interesting dynamic behaviour often occurs over very long timescales – potentially decades. To make this complexity scientifically digestible, different simplified models have been developed for biological networks, as well as semi-empirical laws. A simplified model is useful for exploring the complex behaviour of these biological networks, and to provide greater understanding of experimental data by eliminating the effects of finite sample size and providing precise control over the conditions. These models can then be used to understand macroscopic or mechanical properties of the networks, either directly or to parameterise multiscale approaches.^6,20^ Examples of existing simplified graph-based models include Erdös–Rényi random graphs in which edges are randomly placed connecting nodes, Mikado networks for many-layered systems in which edges are randomly placed across one another, and bond switching in which edges in an ordered graph are randomly exchanged.^21,22^

In this work, an extension of a bond switching method, known as the Wooten–Winer–Weaire (WWW) algorithm, is presented to generate 2D networks which align with those observed in biological systems.^23,24^ Bond switching methods offer good control over physical properties in the networks generated, and are sufficiently flexible to reproduce a wide range of networks. To rigorously compare networks generated by bond switching to real biological networks, key metrics are extracted from a range of experimental microscopy images of networks made of collagen IV or laminin.

Bond switching is a stochastic sampling (often described as a Monte Carlo) method, in which an initially regular network is systematically modified by a series of edge deletion and addition moves (corresponding to bond-breaking and bond-creation in chemical networks). In the WWW method,^24^ the moves are chosen randomly to “switch” the coordination arrangements around two nodes. These switches preserve the coordination number *k*, of each node. For a more detailed discussion of the original method and figures, see Wooten *et al.*^24^ However, in the extended bond switching method, the mean coordination number 〈*k*〉 is fixed at an initial value whilst the *k* of individual nodes may change. A schematic image of this is available as Fig. SI 1 in the ESI.† The changes in *k* are affected by the potential model and the moves chosen. The relaxation of the move selection criterion is especially useful for generating biological continuous random networks as it can encompass a range of different behaviours, for example cross-linking along the backbone of biopolymers or lateral interactions of head groups.

The steps in our algorithm are:

• Initialise using a network with known polygon structure, calculating the energy according to a potential model.

• Select one edge between two nodes and make a trial move by deleting it, and adding a new edge between the first node and a neighbour of the second.

• Locally optimise the geometry around the switching site according to the potential model and recalculate the energy (giving the energy difference Δ*E*).

• Accept or reject the trial move randomly with probability *p*, determined by the Metropolis criterion:^25^

1 *p* = min[1,exp(−Δ*E*/*k*_B_*T*)]

• If the trial move was rejected, return to the previous accepted state. If it was accepted, replace the network with the trial network and repeat the process.

This algorithm is quick to compute, because the geometry only needs to be optimised in a small region around the switching site. The algorithm works best for potential models with explicit bonds and well-defined neighbours. To this end, a simplified Keating potential is applied which consists of harmonic bond (of length *r*) and angular (of angle *θ*) terms:^26^2
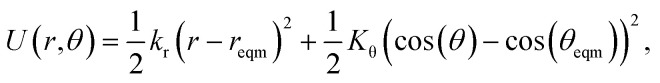
with the equilibrium bond length *r*_eqm_ fixed at 1, and the equilibrium angle *θ*_eqm_ chosen to be 
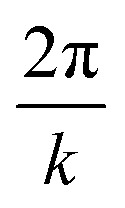
 with *k* the coordination number of the central node. The force constants *k*_r_ and *K*_θ_ are both free parameters. When the coordination number of two nodes changes in the simulation, the *θ*_eqm_ is recalculated for both of them. Fixing *θ*_eqm_ (*i.e.* not changing with *k*) led to the final networks exhibiting more artefacts and often showing significant demixing of nodes.

This simple, but physically motivated, potential model is useful to explore the range of networks that can be generated. Future developments will show how more detailed and biologically accurate potential models, such as the exponential–quadratic form used by Burd,^27^ may further improve agreement with experimental observation. The simplicity of the potential model could be useful as a framework to be combined with more complex requirements. For example, a strong energetic penalty could be applied to configurations exhibiting undesirable artefacts. In this work this technique is used to reject all configurations in which any node has coordination number *k* ≥ 8 or a polygon with side count *n* ≥ 20. Ormrod Morley and Wilson extend this idea further; by using Monte Carlo to minimise a cost function instead of energy directly they demonstrate how key structural metrics (there the fraction of 6-membered rings in amorphous graphene) can be targeted.^23^ It is, however, useful to develop an understanding of the structures formed by simplistic models prior to considering how to effectively contrain them to eliminate unphysical properties, rather than pre-judging what is required.

A honeycomb network of hexagons with 〈*k*〉 = 3 is used as the starting lattice, to allow comparison with previous networks.^28,29^ The use of a well-defined initial polygon structure, and monitoring the changes at each step, allows the algorithm to ensure that the structure generated is physically acceptable. By constraining the mean node coordination, the mean number of edges per polygon is also constrained because of Euler's characteristic equation for polyhedra. For a 2D network which is either periodic or infinitely large, 〈*k*〉 = 3 corresponds to 〈*n*〉 = 6. This does not hold for finite-size aperiodic networks, such as those typically imaged experimentally. However, the approximation for aperiodic systems is small (for example, for 100 rings, 〈*n*〉 = 5.94).^16^

The biological networks considered are visually extremely diverse. To demonstrate that diversity, three networks are shown in Fig. 1, which are accompanied by snapshots of the simulated networks, showing that these can qualitatively replicate the range of observed structures. The more regular networks in the figure are characteristic of those formed by inorganic materials.^16^ The figure also highlights associated structural metrics, the second moment of the ring distribution, *μ*_2_(*k*), and the assortativity, *r*, both of which are discussed below. The difference in accompanying structural metrics in the experimental and simulated configurations highlights the need for a quantitative comparison instead of qualitative similarity. The structural metrics were extracted by a combination of image analysis tools and a mathematical approach based on graph theory as described below. A microscope image can be converted into an abstract graph by extracting the positions of edges and nodes, here using the Ridge Detection plugin for the image processing package ImageJ.^30,31^ The image analysis algorithms are discussed in detail in the ESI.† Obtaining a set of nodes and edges for each image allows the same analysis methods to be applied for the simulated networks.

**Fig. 1:**
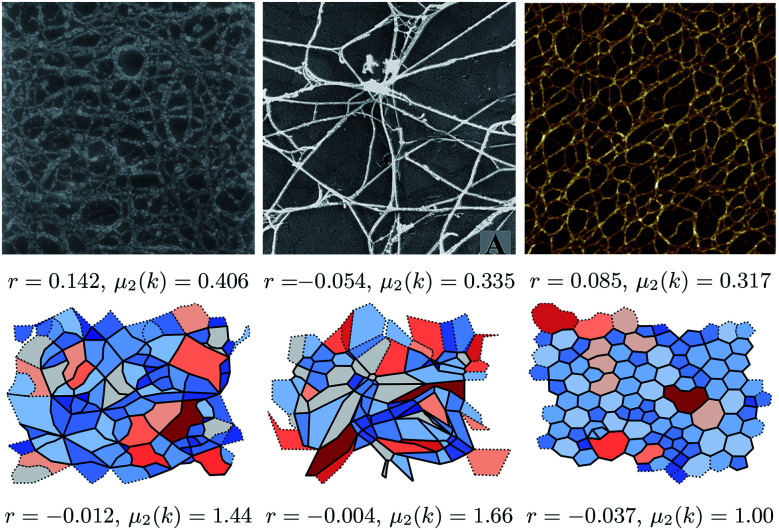
Images of biological network from (left to right) Barnard *et al.*,^32^ Bos *et al.*,^33^ and Wang *et al.*^34^ respectively. The lower panels show networks generated by our method that appear similar, from left to right with {*k*_l_, *K*_θ_} = {0.01, 0.01}, {10, 0.01} and {100, 100}. The simulated networks were chosen on grounds of visual similarity; the difference in accompanying metrics (assortativity *r* and second moment of the *k* distribution *μ*_2_(*k*)) demonstrates why a more rigorous method of comparison is needed. The structural metrics are discussed in more detail in the body of the text. Reproduced from Barnard *et al.*^32^ and Bos *et al.*,^33^ Copyright (1992) and (2001), with permission from Elsevier. Reproduced from Wang *et al.*,^34^ Copyright (2017) with permission.

Images of biological networks produced by Barnard *et al.*, Bos *et al.*, Wang *et al.*, and Yurchenco *et al.* taken between 1984 and 2017 were analysed.^32–36^ A subset of the results are shown in Table 1, and the complete data can be found in the ESI.† The experimental networks show a mean node coordination 〈*k*〉 = 2.78, with a standard deviation of 0.237. The average number of polygon sides 〈*n*〉 is 6.88, with a standard deviation of 1.42. The data seen in Table 1 show slightly lower 〈*k*〉 and larger 〈*n*〉 than for randomised hexagonal networks (where 〈*k*〉 = 3 and 〈*n*〉 = 6).

**Table 1:** Statistics collected for a number of images of biological networks (imaged under different conditions) showing the mean node coordination, 〈*k*〉, the second moment, *μ*_2_(*k*), the mean number of polygon edges, 〈*n*〉, the number of polygons in each configuration, *N*, and the assortativity, *r*. Two different analyses of the networks imaged by Yurchenco *et al.*^35^ are shown in order to highlight the sensitivity of the obtained metrics. A more detailed table is available in the ESI

Image	〈*k*〉	*μ*_2_(*k*)	〈*n*〉	*r*	*N*
Barnard *et al.*^32^	2.961	0.378	5.574	−0.109	135
Bos *et al.*^33^	2.780	0.335	5.825	−0.209	40
Wang *et al.*^34^	2.945	0.805	5.735	−0.057	498
Yurchenco and Furthmayr^35^	2.721	0.284	6.254	−0.181	61
Yurchenco and Furthmayr^35^	2.667	0.287	6.593	−0.184	54

However, some caution is required in the interpretation of the results obtained with these methods. Fig. 2 demonstrates the difficulty in assigning edges to an image. The experimental image (left panel, from Yurchenco and Furthmayr^35^) is analysed using two different manual interpretations of the network. As the assignment of the edges can be ambiguous, there are many equally valid ways to select which edges are retained, and so different polygon structures arise. Here, for example, a small change in the algorithm identifies more nodes with a small associated increase in the mean node coordination, 〈*k*〉, and reduction in the mean polygon side count 〈*n*〉 (Table 1). Variations in 〈*k*〉 are emphasised by an ambiguity as to whether an edge in a biological network is best described as being one biomolecule or two, which makes describing the fraction of 2-coordinate nodes problematic. 〈*k*〉 is also affected by the finite size of an image, because where an edge is terminated by the image boundary, we remove it from the calculations, reducing *k* for edge nodes. Missing edges, either from low contrast or when terminated by an edge, leads polygons to be counted as too large or to not be counted at all respectively. These issues are more significant in smaller or lower-detail images, because the contrast leads to worse ring identification and a greater fraction of polygons are on the boundary.

**Fig. 2:**
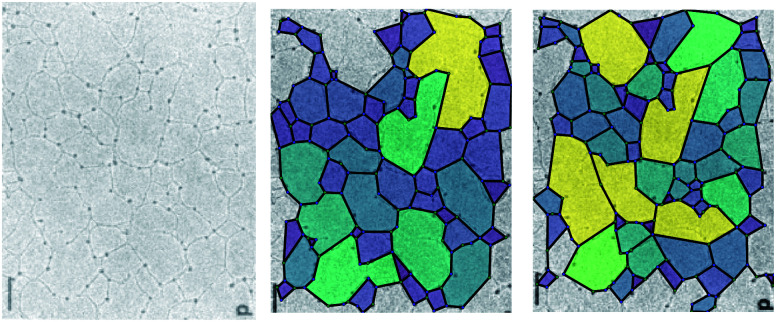
The polygon structure of a collagen IV basement membrane as imaged by Yurchenco and Furthmayr.^35^ The two images are different ways of highlighting the polygons in the same image, according to which ambiguous edges are included. Reprinted (adapted) with permission from Yurchenco and Furthmayr.^35^ Copyright 1984 American Chemical Society.

The data extracted from the experimental images (Table SI 1 in the ESI†) indicate that the most useful metrics for describing these networks are the width of the node distribution (characterised by the second moment, *μ*_2_(*k*)), and the assortativity *r*, as described by Newman.^15^ For hexagonal inorganic networks, *μ*_2_(*k*) = 0, whereas all of the simulated and imaged networks have 0.3 ≲ *μ*_2_(*k*) ≲ 1.2. The assortativity represents the similarity of neighbouring polygons, and is bounded as −1 ≤ *r* ≤ 1 with a negative value corresponding to polygons being neighbours to dissimilar polygons, and positive values indicating similar neighbours. An assortativity of *r* = 0 means that there is no preference for similar or dissimilar neighbours, and is seen in Erdös–Renyi random graphs. If the assortativity were ruled by geometrical constraints alone in a random point process, we would expect *r* ≈ −0.15.^16^ Hard-disk simulations of colloids have shown positive assortativities of ∼0.4,^38^ networks of biological reactivity have small negative assortativities,^15^ whilst inorganic networks show negative values whose magnitudes depend on the details of the building blocks.^16^

Although the polygon side count distributions (characterised by the mean number of sides, 〈*n*〉 and its second moment, *μ*_2_(*n*)) have been extensively used to characterise more regular networks (see, for example, Ormrod Morley and Wilson^23^ and references therein) the greater disorder inherent in the networks considered here makes these measures more difficult to assess. For example, 〈*n*〉 is highly sensitive to finite size effects, whilst *μ*_2_(*n*) appears equally sensitive to the presence of (rare) large polygons. Fig. 3(a) shows the variation of *r* as a function of *μ*_2_(*k*) for the experimental networks, an effective “network landscape”. The experimental networks show significant range of {*μ*_2_(*k*), *r*} values, with the majority (14 of 19) in the range −0.25 ≤ *r* ≤ 0. There is a weak correlation between the two metrics with *r* becoming less negative as *μ*_2_(*k*) increases (see below).

**Fig. 3:**
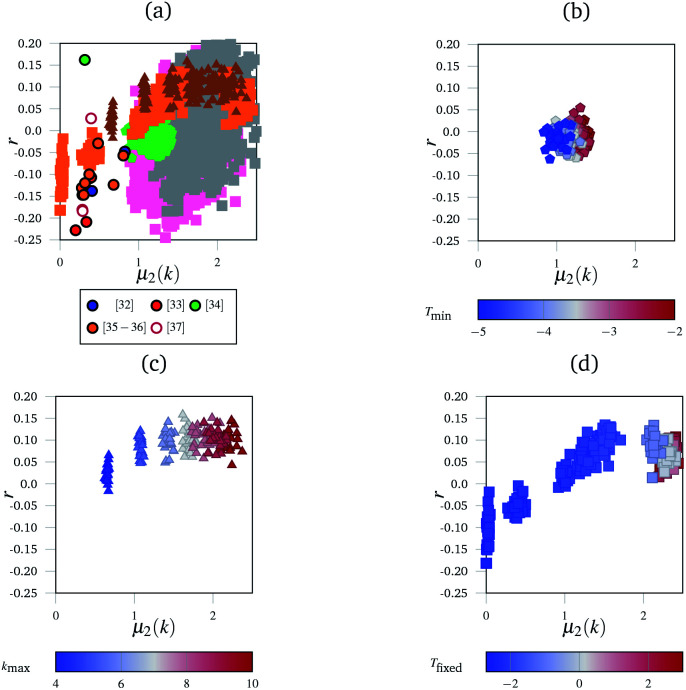
The “network landscape” showing both the simulated and experimental networks. In subfigure (a) different markers represent sets of simulated results, and outlined circles represent experimental data from the references indicated.^32–37^ Grey and pink squares represent the networks generated by the simulated annealing method with different bond and angle strength parameters. The subfigures highlight subsections of that landscape under different conditions with consistent markers to subfigure (a)—for example, triangles in subfigure (a) represent the same data as the triangles in subfigure (c). The subfigures are coloured according to which parameter was varied in the simulation. In (b), this is the minimum temperature *T*_min_ the simulation reached. In (c), this is the maximum coordination number of an individual node *k*_max_. In (d), it was the fixed temperature *T*_fixed_ the networks were heated at.

To develop a systematic understanding of the range of networks formed, networks were generated using a wide range of potential model parameters. Network configurations were generated with *k*_l_ and *K*_θ_ ranging from 0.01 to 100 reduced units, changing from networks dominated by bond strain to those dominated by angle strain. The main method of network generation was simulated annealing. First, the networks were thermalised at very high temperature (corresponding to 1 × 10^4^ temperature units), effectively accepting all physical Monte Carlo moves. The thermalisation continued for 10 000 steps at that high temperature, which led to complete melting. After thermalisation, the networks were cooled. The cooling proceeded by making *n* moves at a temperature *T*, then reducing *T* by a constant multiplicative factor and making another *n* steps. This was repeated until a pre-set minimum temperature was reached. One example simulation had 1000 moves made at *T* = 1 × 10^4^, then a further 1000 moves at *T* = 1 × 10^3.8^, and so on until *T* = 1 × 10^−2^. The logarithmic nature of the temperature scaling was necessary to access the wide range of temperatures between the melted and frozen states in a computationally efficient manner.

Fig. 3 shows the range of different networks which can be generated. There is a great deal of uncertainty around each experimental point, owing to the ambiguity previously discussed. Fig. 3(b)–(d) show the evolution of *r* as a function of *μ*_2_(*k*) for the simulated networks generated under different conditions.

Fig. 3(b) shows the effect of cooling the networks for different lengths of time, but at the same rate. Networks that were cooled for longer are in blue, and it shows a reversal of the entropy driven effects evident in Fig. 3(b). This demonstrates the sensitivity of the networks to the annealing procedure. As networks are cooled for longer, the assortativity remains broadly unchanged, but the node distribution histograms become narrower; the highly coordinated nodes were enthalphically unfavourable because of the angular strain they induced in the nodes around them.

Finally, Fig. 3(c) shows the effect of limiting the maximum permissible node coordination to be in the range 2 ≤ *k* ≤ *k*_max_. For a low *k*_max_ = 4, the networks have narrow node distributions and polygon assortativities close to the random limit. However, as *k*_max_ increases to 10, the widths of the node distributions increase and the networks become assortative. Nodes with large *k* were often surrounded by narrow triangles to accommodate the angular strain. Since there are *k* neighbouring triangles around each node this leads to a positive assortativity.

Fig. 3(d) shows networks generated using an alternative method to the simulated annealing protocol described above. Here, networks are partially thermalised at a range of fixed temperatures with fixed potential parameters *k*_l_ and *K*_θ_. At low temperatures the networks formed are highly correlated (disassortative) whilst at higher temperatures the networks formed are mildly assortative, corresponding to a partial demixing. There are two main areas in this landscape, found at the bottom-left (negative *r* and low *μ*_2_(*k*)) and the top right (positive *r* and high *μ*_2_(*k*)). The bottom-left region is occupied by networks generated at low temperatures, and corresponds to the presence of uncorrelated defects in the regular lattice structure. The defects are similar to those described as Stone–Wales defects in carbon networks, and since they are surrounded by ordered structures this leads to disassortative mixing.^39^ The concentration of independent defects is a function of temperature, and hence an increased temperature leads to a larger *μ*_2_(*k*) as more defects appear. At higher temperatures, in the top-right region, the networks are dominated by entropy. The width of the node distribution function increases to a maximum, which is discussed below, and the networks end up close to the random limit.

Fig. 3(a) shows the combined metrics from Fig. 3(b)–(d) along with the full sets of results obtained by varying *k*_l_ and *K*_θ_. The experimental data, taken from ref. 32–37, lies at relatively low *μ*_2_(*k*) and negative *r*. This low *μ*_2_(*k*) and negative *r* region is occupied by relatively few simulated networks, with most simulated networks showing a larger *μ*_2_(*k*) value. This is a combination of multiple factors that were explored in the panels of Fig. 3. Highly coordinated tangles such as those seen experimentally in Fig. 1(b) are relatively rare, and node coordinations above 4 are geometrically unstable with respect to splitting into two 3-coordinate nodes. This naturally limits the width of the node distribution histograms, and hence *μ*_2_(*k*). Next, many biological networks are constructed in an ordered manner, or enthalpically driven in their assembly. For example, collagen IV in basement membranes has been hypothesised to have a square or a regular hexagonal structure.^27,40^ The images analysed here could be of regular networks with defects, either from aging or from preparation for imaging. Finally, the position of experimental data shows that real networks are not close to the random limit. This matches up with our intuition, as maximised entropy limits the amount of useful work that can be extracted from a system, which is an unhelpful property for biological networks. We anticipate that future network models which take the enthalpic factors into account more accurately (such as having terms in the potential that disfavour demixing) will fill in the “valley” observed in the landscape.

The simulated networks show interesting physical properties, including demixing, which are not observed in the experimental images. Similar polygons tended to cluster together and become more convex as the networks were cooled for longer periods of time. This demixing is driven enthalpically, as it appears stronger at low temperatures. The enthalpic drive arises from accommodating angular strain, for example, four-coordinate nodes are least strained when in a region of squares, and two-coordinate nodes are least strained when they are part of a long boundary between two large rings. After demixing, the networks show a positive assortativity because of this strain-driven preference for similar shapes to be adjacent. The potential slow evolution of structure has significant implications for the network aging process. This could be important, for example, in the collagen-IV network in the eye.

In conclusion, the bond switching method developed here can generate 2D polygonal networks similar to those found in biology. It can generate networks with a wide range of polygon side counts and node coordination distributions, mirroring the diversity exhibited by biological networks. A number of experimental biological networks were studied using methods previously used for inorganic chemical networks, and techniques from network theory were applied to understand their short range structure. The biologically-inspired networks showed the importance of letting the node distribution vary, and were almost all disassortative. However, the simulated networks show a range of assortativities approximately in the range −0.2 ≤ *r* ≤ 0.2. This is an interesting result, as the extra degree of freedom allowed in the simulations by varying *k* allows the polygon networks to get closer to the maximum entropy solution (with *r* ≈ 0), which is not possible in traditional bond switching approaches.^16^ The enthalphic drive towards disassortative mixing in real networks is an interesting topic, and could be studied in future by improvements to the simplified potential model presented here. Analysing these biological networks presented challenges because of the ambiguity in identifying edges, and it is anticipated that the simulated networks can be used to help resolve this. The simulated networks can bridge the gap between the hexagonal networks favoured by inorganic materials and more disordered biological networks, as well as showing interesting physical properties of their own, such as demixing. The additional degree of freedom allows for more entropically-dominated polygon networks, which allows the assortativity to approach zero. Future work will build on these methods utilising improved potentials, and exploring further the role of entropy, in order to understand the factors which underpin the observed network landscape.

## Conflicts of interest

There are no conflicts to declare.

## Supplementary Material

RA-010-D0RA06205G-s001

## References

[cit1] Ormrod Morley D., Wilson M. (2019). Mol. Phys..

[cit2] Weaire D., Rivier N. Y. (2009). Contemp. Phys..

[cit3] Le Caër G., Delannay R. (1993). J. Phys. A: Math. Gen..

[cit4] Zachariasen W. H. (1932). Phys. Rev..

[cit5] Hartwig J. H., Yin H. L. (1988). Cell Motil. Cytoskeleton.

[cit6] Burd H. J. (2009). Biomech. Model. Mechanobiol..

[cit7] Hamill K. J., Kligys K., Hopkinson S. B., Jones J. C. (2009). J. Cell Sci..

[cit8] Kang B., Jo S., Baek J., Nakamura F., Hwang W., Lee H. (2019). Acta Biomater..

[cit9] Schliecker G., Klapp S. (1999). Europhys. Lett..

[cit10] Aboav D. A., Langdon T. G. (1969). Metallography.

[cit11] Wright A. C., Thorpe M. F. (2013). Phys. Status Solidi B.

[cit12] Lewis F. T. (1926). Anat. Rec..

[cit13] Gervois A., Troadec J. P., Lemaitre J. (1992). J. Phys. A: Math. Gen..

[cit14] Aboav D. A. (1980). Metallography.

[cit15] Newman M. E. J. (2002). Phys. Rev. Lett..

[cit16] Ormrod Morley D., Thorneywork A., Dullens R., Wilson M. (2020). Phys. Rev. E.

[cit17] Boerboom R. A., Krahn K. N., Megens R. T., van Zandvoort M. A., Merkx M., Bouten C. V. (2007). J. Struct. Biol..

[cit18] Barlow A. M., Mostaço-Guidolin L. B., Osei E. T., Booth S., Hackett T.-L. (2020). PLoS One.

[cit19] Reese S. P., Farhang N., Poulson R., Parkman G., Weiss J. A. (2016). Biophys. J..

[cit20] Sander E. A., Stylianopoulos T., Tranquillo R. T., Barocas V. H. (2009). Proc. Natl. Acad. Sci. U. S. A..

[cit21] Broedersz C. P., Mackintosh F. C. (2014). Rev. Mod. Phys..

[cit22] Picu R. C. (2011). Soft Matter.

[cit23] Ormrod Morley D., Wilson M. (2018). J. Phys.: Condens. Matter.

[cit24] Wooten F. O., Winer K., Weaire D. (1985). Phys. Rev. Lett..

[cit25] Metropolis N., Rosenbluth A. W., Rosenbluth M. N., Teller A. H., Teller E. (1953). J. Chem. Phys..

[cit26] Kuronen A., Kaski K., von Alfthan S. (2003). Phys. Rev. B: Condens. Matter Mater. Phys..

[cit27] Burd H. J., Regueiro R. A. (2015). Biomech. Model. Mechanobiol..

[cit28] Barkema G. T., Mousseau N. (2000). Phys. Rev. B: Condens. Matter Mater. Phys..

[cit29] Limbu D. K., Atta-Fynn R., Biswas P. (2019). MRS Adv..

[cit30] Schneider C. A., Rasband W. S., Eliceiri K. W. (2012). Nat. Methods.

[cit31] Wagner T. , Hiner M. and Raynaud X., Ridge Detection, 2018, https://github.com/thorstenwagner/ij-ridgedetection

[cit32] Barnard K., Burgess S. A., Carter D. A., Woolley D. M. (1992). J. Struct. Biol..

[cit33] Bos K. J., Holmes D. F., Meadows R. S., Kadler K. E., McLeod D., Bishop P. N. (2001). Micron.

[cit34] Wang Z., Xiao Q., Song X., Wan Y., Zhu J. (2017). J. Food Qual..

[cit35] Yurchenco P. D., Furthmayr H. (1984). Biochemistry.

[cit36] Yurchenco P. D., Ruben G. C. (1987). J. Cell Biol..

[cit37] Fabris G., Lucantonio A., Hampe N., Noetzel E., Hoffmann B., DeSimone A., Merkel R. (2018). Biophys. J..

[cit38] Chremos A., Camp P. J. (2007). Phys. Rev. E.

[cit39] Stone A. J., Wales D. J. (1986). Chem. Phys. Lett..

[cit40] Cummings C. F., Pedchenko V., Brown K. L., Colon S., Rafi M., Jones-Paris C., Pokydeshava E., Liu M., Pastor-Pareja J. C., Stothers C., Ero-Tolliver I. A., McCall A. S., Vanacore R., Bhave G., Santoro S., Blackwell T. S., Zent R., Pozzi A., Hudson B. G. (2016). J. Cell Biol..

