# Supplementary material for: Simplified computational model for generating biological networks
Source: RSC Adv. 2020 Oct 16;10(63):38275–80. doi: 10.1039/d0ra06205g (PMC9057274; doi:10.1039/d0ra06205g)
Supplement: RA-010-D0RA06205G-s001 [file RA-010-D0RA06205G-s001.pdf]

Electronic Supplementary Material (ESI) for RSC Advances.
This journal is © The Royal Society of Chemistry 2020

Simplified Computational Model for Generating Biological Networks Electronic Supplementary Information

Matthew H J Bailey,* David Ormrod Morley,* and Mark Wilson*

SI I Image Analysis Algorithm

SI I.1 Image to Graph

In this work, we describe an image analysis algorithm to extract polygon statistics from experimental microscope images. This algorithm relies on image processing software, ImageJ[30], and specifically the Ridge Detection plugin. As the images show very different contrast levels, colourations and resolution, each image had to be manually assessed in order to extract reliable data. We anticipate that this problem will be eased if the process were applied to a consistent set of images from a single experiment. Where edges were unclear in images and could not be detected by ImageJ, we manually highlighted them. The output consisted of node coordinates and their associated edge connectivity. The next step of the algorithm wass to fit edges in a graph to each line segment. This was performed using an orthogonal least squares regression on each line segment to find the best straight line fit. Each line segment was placed in a graph as a pair of nodes connected only to each other, and the nodes were associated with the end positions of the line segments in the planar embedding. Next, nearby groups of nodes were connected; if two nodes were within a cutoff (usually chosen to be in the range 5-10 pixels, depending on the scale and resolution of the image) they were grouped into a single node cluster. This process was repeated along a chain. For example, if node a and node b were within the cutoff, and node c was within a cutoff of b but outside the cutoff of a, they would all be clustered into abc. These clusters preserved the connections of each of the line segments included, until a connected graph was formed.

SI I.2 Graph to Polygons

With a connected graph formed, we had to extract the polygon data. We did this by removing all nodes with $k = 1$ (including nodes on the boundaries of the image) repeatedly until no nodes with $k = 1$ existed. Next, we constructed the Delaunay triangulation of the points, which was a superset of the connected graph for all the systems we tested. The simplices in the Delaunay triangulation are key to identifying the polygon structure. We one-by-one removed the edges in the Delaunay triangulation that were not present in the original graph. Where removing an edge joined two polygons, we tracked that and marked them as a single polygon. When the process had removed all edges that were not in the original connected graph, we had a list of all the polygons in the embedded graph.

This avoids problems with previous definitions of polygon structure as, owing to the Delaunay triangulation, there is only one unambiguous assignment of these polygons. Finally, we constructed a dual graph, with the nodes being placed at the centroids of each ring and the edges representing two rings sharing one or more edges. We used this dual graph to calculate the assortativity of the ring network.

SI II Data

We extracted data from images produced by Barnard *et al.*[32], Bos *et al.*[33], Wang *et al.*[34], Yurchenco and Furthmayr[35], Fabris *et al.*[37] and Yurchenco and Ruben[36] The full data are available here in Table SI 1.

Department of Chemistry, Physical and Theoretical Chemistry Laboratory, University of Oxford, South Parks Road, Oxford OX1 3QZ; E-mail: mark.wilson@chem.ox.ac.uk
* these authors contributed equally to this work.

Author	Figure	$\langle k\rangle$	$\mu_2(k)$	r_k	$\langle n\rangle$	$\mu_2(n)$	r_n	N_{node}	N_{Polygon}	$p_{k,3}$	$p_{n,6}$
Barnard *et al.*	2	2.525	0.406	0.142	6.989	24.634	-0.138	385	91	0.385	0.077
Barnard *et al.*	4 c	2.961	0.378	-0.071	5.574	4.113	-0.109	283	135	0.696	0.185
Barnard *et al.*	6	3.154	0.824	0.012	5.122	3.602	-0.048	520	278	0.529	0.169
Bos *et al.*	2 a	2.780	0.335	-0.054	5.825	7.892	-0.209	100	40	0.620	0.225
Bos *et al.*	5 c	2.884	0.198	0.171	5.308	4.324	-0.228	86	39	0.791	0.128
Bos *et al.*	5 d	2.974	0.396	0.188	5.385	6.461	-0.107	228	109	0.632	0.119
Fabris *et al.*	3 f	2.335	0.315	-0.108	5.893	15.210	0.162	155	19	0.246	0.316
Fabris *et al.*	4 b	2.365	0.285	-0.077	7.043	27.154	-0.147	230	32	0.313	0.094
Wang *et al.*	1 c	2.657	0.681	0.064	7.250	125.990	-0.124	621	185	0.353	0.081
Wang *et al.*	2 a	2.534	0.372	-0.027	8.497	31.807	-0.100	618	163	0.416	0.153
Wang *et al.*	2 d	2.867	0.277	-0.022	5.545	3.021	-0.131	150	66	0.727	0.258
Wang *et al.*	3 b	2.945	0.805	-0.019	5.735	15.342	-0.057	1071	498	0.458	0.122
Wang *et al.*	4 a	3.006	0.317	0.085	5.520	5.391	-0.120	348	173	0.684	0.139
Wang *et al.*	5 b	2.531	0.301	-0.031	8.755	27.563	-0.147	350	94	0.480	0.128
Wang *et al.*	5 c	2.991	0.484	0.231	5.364	4.601	-0.029	216	97	0.611	0.175
Yurchenco and Furthmayr 1984	4 b alternative	2.721	0.284	0.074	6.254	6.999	-0.181	172	61	0.657	0.213
Yurchenco and Furthmayr 1984	4 b	2.667	0.287	-0.033	6.593	10.510	-0.184	159	54	0.604	0.241
Yurchenco and Ruben 1987	1 c	2.792	0.395	-0.028	5.093	2.610	0.028	106	42	0.595	0.095
Yurchenco and Ruben 1987	2 f	2.696	0.303	0.170	5.880	6.443	-0.363	69	25	0.610	0.200

Table SI 1 A full table of the experimental data, with the mean node coordination $\langle k\rangle$, the second moment of the node coordination distribution $\mu_2(k)$, assortativity of nodes r_k, mean polygon size $\langle n\rangle$, the second moment of the polygon size $\mu_2(n)$, assortativity of polygons r_n, number of nodes and polygons N_{Node} and N_{Polygon}, as well as the fraction of 3-coordinate nodes and 6-members polygons $p_{k,2}$ and $p_{n,6}$. Data is taken from Barnard *et al.*[32], Bos *et al.*[33], Fabris *et al.*[37], Wang *et al.*[34], and Yurchenco and Furthmayr[35], Yurchenco and Ruben[36].

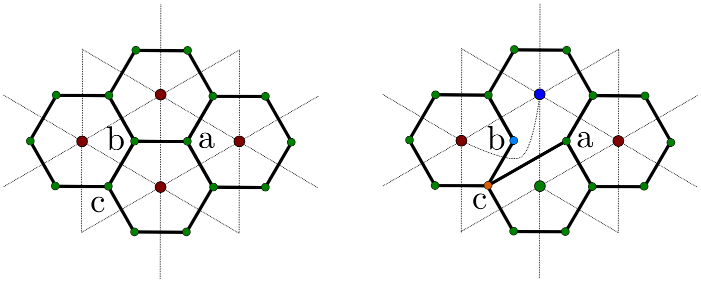

Fig. SI 1 The bond between edge a and b is switched to be between edges a and c. After the switch, node c is four coordinate and node b is two coordinate, with all others being three coordinate. Finally, the network geometry is optimised to a local minimum of the potential.

SI III Bond switching.

Figure SI 1 shows a schematic of the bond switching algorithm described in the main text. The solid lines represent real edges in the graph, and the dotted lines represent edges in the dual graph, which connects the centres of polygons. The edge between a and b is switched to be between a and c. This changes the coordination numbers of b from 2 to 3, and c from 3 to 4, preserving the average coordination number $\langle k\rangle = 3$. The number of polygon edges (represented as coordination in the dual graph) changes from $\{6,6,6,6\}$ to $\{5,6,6,7\}$.
